# CT Imaging Findings of Pulmonary Artery Stenosis: A Pictorial Review

**DOI:** 10.3390/diagnostics14161762

**Published:** 2024-08-13

**Authors:** Mengdi Zhang, Li Chen, Chao Bu, Hanxi Zhang, Jing Luo, Jing Wang, Qihua Sun, Qingyu Liu, Zhonghua Sun, Yu Li

**Affiliations:** 1 Department of Radiology, The Seventh Affiliated Hospital of Sun Yat-sen University, Shenzhen 518107, China; zhangmd27@mail.sysu.edu.cn (M.Z.); buch3@mail.sysu.edu.cn (C.B.); 5tlai@163.com (H.Z.); luojing923@163.com (J.L.); jun2758an@163.com (J.W.); sunqihua@sysush.com (Q.S.); liu.qingyu@163.com (Q.L.); 2 Department of Neurology, Shenzhen Luohu People’s Hospital, Shenzhen 518107, China; chen2power@163.com; 3 Discipline of Medical Radiation Science, Curtin Medical School, Curtin University, Perth 6845, Australia; 4 Curtin Health Innovation Research Institute (CHIRI), Curtin University, Perth 6845, Australia

**Keywords:** pulmonary artery stenosis, congenital and acquired anomalies, computed tomography pulmonary angiography, differential diagnosis

## Abstract

Pulmonary artery stenosis represents a group of disorders involving main, branch or peripheral pulmonary arteries with pain, dyspnea, hemoptysis or even no symptoms. Early diagnosis and timely intervention are crucial for reducing mortality, but timely diagnosis is challenging due to the non-specific symptoms. Computed tomography pulmonary angiography (CTPA) is useful in the diagnosis because it can provide more details about abnormal changes in the lumen, vessel wall and adjacent mediastinal structures. Congenital and acquired pulmonary artery anomalies have some characteristics on CTPA, which can be useful for differential diagnosis. Awareness of these conditions is important for radiologists. This pictorial review provides an overview of CTPA imaging features of pulmonary artery stenosis.

## 1. Introduction

Pulmonary artery stenosis (PAS) is caused by the formation of obstructive lesions in the pulmonary artery and its branches. Due to the decreased cross-sectional area of the pulmonary artery, blood flow exiting the heart can be impeded. This may raise the pressure in the right ventricle (RV), causing endothelial damage to the PA and further leading to pulmonary artery hypertension (PAH). Among all the causes, right heart failure can lead to poor prognosis and even death [1].

PAS can be categorized based on location, and there are four types, from proximal to subsegmental. Type I involves single central stenosis of the main pulmonary trunk or branches of the pulmonary artery. Type II involves the bifurcation of the pulmonary artery. Type III involves multiple peripheral stenosis. Type IV involves a combination of both main and peripheral stenosis [2].

The clinical presentation of PAS varies from shortness of breath, fatigue and tachycardia to swelling of the feet, ankles and abdomen, which happens in the advanced stage due to right heart failure. Thus, detecting PAS as early as possible is important for improving the prognosis.

When a patient presents with the previously described symptoms, chest radiography or computed tomography should be conducted. High-resolution computed tomography (HRCT) and computed tomography pulmonary angiography (CTPA) are invaluable for examining the pulmonary vessels, which can provide details of the lumen, vessel wall, adjacent mediastinal structures and subsequent lung lesions, which are very important for identifying the causes of PAS.

The aim of this article is to review the imaging features of congenital and acquired anomalies that cause pulmonary artery stenosis, and to illustrate the typical CT characteristics of these anomalies. We will discuss the congenital diseases causing a decrease in right ventricular output volume, as well as pulmonary artery and pulmonary vein atresia. For simplicity, the acquired diseases will be categorized as anomalies affecting the vessel wall, intraluminal anomalies and extraluminal anomalies. A table of contents of these diseases categorized by the causes of PAS is provided (Table 1).

## 2. Subjects and CT Scanning Parameters

We retrospectively reviewed 28 patients who were diagnosed with PAS and treated at the Seventh Affiliated Hospital of Sun-Yat Sen university in Shenzhan, China, all of whom underwent HRCT and CTPA between March 2022 and February 2024. On CTPA, pulmonary artery stenosis can be classified into localized and diffused stenosis. Stenosed vessels with localized stenosis were compared with adjacent unaffected vessels, while diffused stenosis was compared with the pulmonary arteries of the contralateral lung. Two consultant radiologists (with 5 and 20 years of experience in cardiac CT imaging, respectively) analyzed the images on 2D axial, multiplanar reconstruction (MPR) and maximum-intensity projection (MIP) views.

All CTPAs were performed on a 192-slice dual-source CT scanner (Siemens Somatom Force, Siemens Healthcare, Forchheim, Germany). The following image acquisition parameters were used: 104 mAs with automatic tube current modulation, pitch 1.7, rotating time 0.25 s and reconstruction slice thickness 1 mm with reconstruction interval 0.7 mm. In total, 50–60 mL of contrast agent was administered at a 4.5 mL/s injection speed, followed by injection of normal saline at the same speed. Dual-phase CTPA was conducted. After the right atrium reached the monitoring threshold of 60 HU, the intelligent tracking method started the first pulmonary artery phase in 2 s, and the second aortic phase was subsequently scanned in 5 s.

## 3. PAS Due to Congenital Etiology

### 3.1. Pulmonary Valvular Stenosis

Valvular pulmonary stenosis is the most common type of congenital pulmonic stenosis, followed by the subvalvular and supravalvular types [3]. It accounts for about 8–10% of all congenital heart disease and contributes to right ventricular outflow obstruction [4]. The high-speed flow through the narrowed valve causes post-stenotic dilatation of the main and left pulmonary arteries [5]. In some cases, due to the decreased blood flow, diffused stenosis of the distal lobular and segmental PA can be detected.

**Figure 1:**
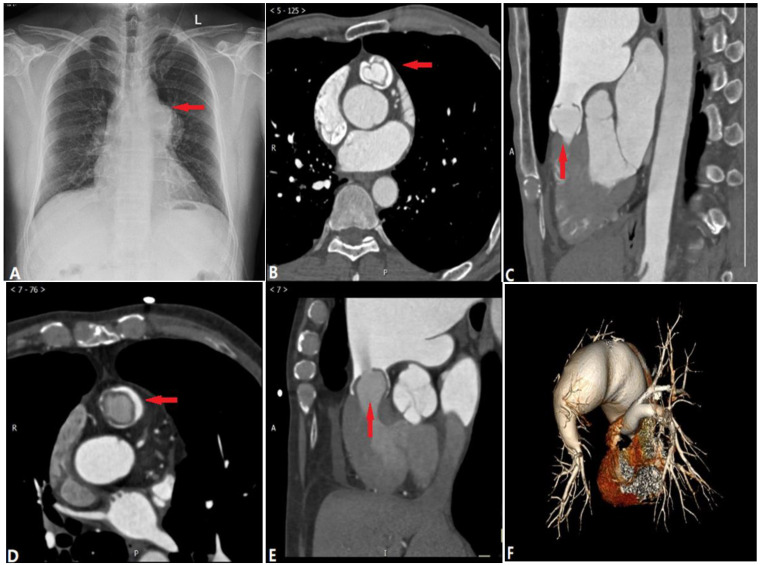
Pulmonary valvular stenosis—bicuspid pulmonary valve. (**A**): Chest radiograph shows the prominence of the left pulmonary artery, while the right pulmonary artery (arrow) and hilum appear normal. (**B**–**E**) belong to two different patients, but both show the stenotic bicuspid pulmonary valve (arrow) and post-stenotic dilation of the main and left pulmonary arteries, whereas the distal branches are relatively small. The jet of blood flow (arrow) is forced through the stenotic valve to the left pulmonary artery in (**E**). (**F**): volume-rendering of pulmonary arteries indicates the post-stenotic dilation of the main and left pulmonary arteries.

On a chest radiograph and CT (Figure 1), the enlargement of the pulmonary trunk and left PA can be seen, but there is a normal right PA, since the left PA is a direct continuation of the pulmonary trunk. Subject to the reduced blood flow of PA and elevated pressure overload of the right ventricle related to the stenotic valve, stenosis of distal PA branches and right ventricular hypertrophy may be visible. A thickened pulmonic valve can also be apparent on ECG-gated computed tomography pulmonary angiography (CTPA) [6].

### 3.2. Pulmonary Artery Atresia

**Figure 2:**
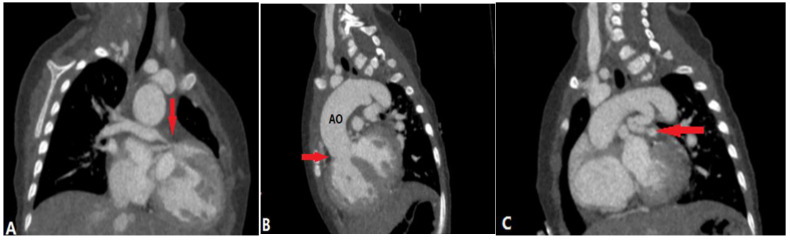
Pulmonary artery atresia with ventricular septal defect. (**A**): Coronal CT angiography shows that the main pulmonary artery is atretic and the right pulmonary artery is slender (arrow). (**B**): Oblique coronal CT shows outlet ventricular septal defect (arrow) and overriding aorta (AO). (**C**): Oblique coronal CT demonstrates patent ductus arteriosus (arrow) originating from aorta to left pulmonary artery.

PA atresia affects 1 out of every 10,000 live births. There are two types of PA atresia, distinguished by the presence or absence of a ventricular septal defect (VSD) [7]. PA-VSD is often a variant of Tetralogy of Fallot (TOF), characterized by discontinuity between the RV and pulmonary arteries, and associated with a VSD (Figure 2). This abnormality includes atresia of the right ventricular outflow tract (RVOT) or pulmonary valve with variable hypoplastic, discontinuous, or absent MPA and central branch PAs. In this case, systemic collaterals must be present to supply the lung, including a patent ductus arteriosus (PDA) or major aortopulmonary collaterals (MAPCAs) from the descending aorta, subclavian, celiac, or coronary arteries. Infants usually present with cyanosis and hypoxia or heart failure [6].

CTPA illustrates the accurate length of pulmonary atresia, the presence of branch PA confluence, the size of the main, right, and left PAs at the origin and hilum, and the pulmonary blood supply, which is necessary for the surgical plan.

PA atresia with intact ventricular septum (PA-IVS) is less common than the former anomaly. The RV is usually hypoplastic and hypertrophied as there is no blood flow through the VSD to promote RV growth, and RV outflow obstruction causes myocardial remodeling. This is different from PA-VSD, in which the RV cavity can grow [6]. In some cases with a dysplastic tricuspid valve, an enlarged RV may be shown. Branch PAs and PDA are frequently normal. Blood flow to the PAs depends on a PDA [7]. Associated abnormalities in the coronary circulation include ventriculocoronary connections, fistulae, coronary stenosis, or atresia.

### 3.3. Pulmonary Vein Atresia

Congenital pulmonary vein atresia (PVA) occurs due to the complete or partial failure of the incorporation of the developmental common pulmonary vein into the left atrium, which is divided into three categories based on the extent of venous involvement [8]. Among these categories, unilateral PVA results in lung hypoplasia and pulmonary artery stenosis [9]. Because PVA occurs after regression of embryological drainage to the systemic cardinal and umbilicovitelline system, an anomalous systemic venous connection is absent. Recurrent respiratory tract infections, hemoptysis, or pulmonary hypertension may be the presenting complaint in unilateral PVA patients.

**Figure 3:**
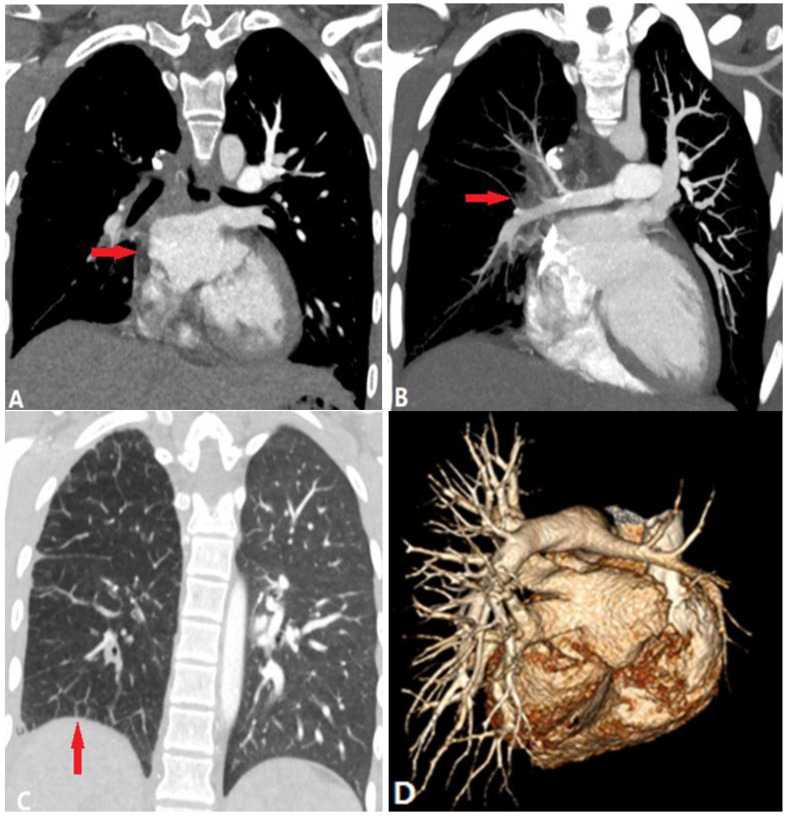
Unilateral pulmonary vein atresia. (**A**): Coronal CTA shows the absence of right pulmonary veins and outpouchings on the left atrium (arrow). (**B**,**C**): MIP and lung windowing coronal CT images show mass-like soft tissue within the right hilum (arrow in (**B**)), septal lines and bronchial-wall thickening (arrow in (**C**)), which indicate the engorged lymphatics and collateral venous channels. (**D**): VR of CTA shows the small caliber of right pulmonary arteries. CTA—computed tomography angiography; MIP—maximum-intensity projection; VR—volume-rendering.

On CTPA, no pulmonary vein drainage to the left atrium is shown in the affected lung, typically without evidence of outpouchings on the left atrium to indicate the rudimentary ostia. Because of the increased pulmonary venous pressure in the ipsilateral lung [9], blood flow is reduced compared to the contralateral side, which is manifested as diminutive pulmonary arteries with diffused ground-glass pulmonary opacities, septal lines, and bronchial-wall thickening. The affected hilum is enlarged due to engorged lymphatics and collateral venous channels (Figure 3).

## 4. PAS Due to Acquired Disease

### 4.1. Intraluminal Anomalies

#### 4.1.1. Pulmonary Thromboembolism (PTE)

**Figure 4:**
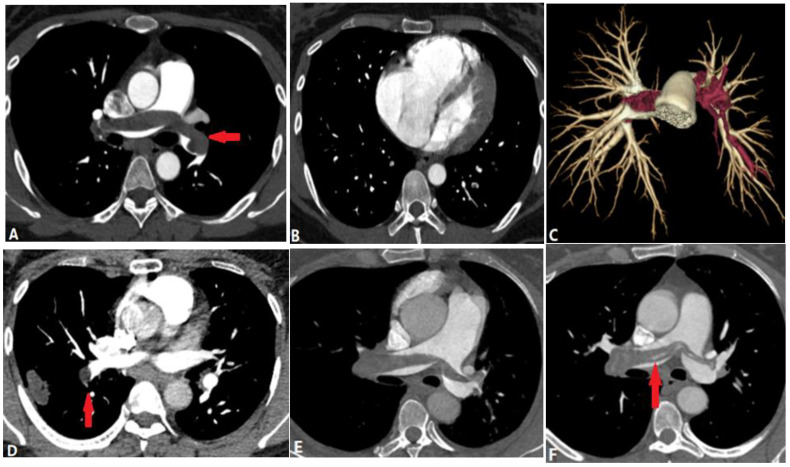
Pulmonary thromboembolism. (**A**): Axial CT image shows a partial filling defect in the left and right pulmonary arteries, which is non-enhanced and surrounded by contrast (arrow). (**B**): Axial CT image shows enlargement of the right ventricle and atrium (RV:LV ratio > 1), and 3D volume-rendering image (**C**) shows the range of the thrombus, which is marked in red. (**D**): Axial CT image shows the complete filling defects of another patient, with no enhancement of the entire lumen (arrow) and subpleural pulmonary infarction. (**E**): Axial CT image of another patient who underwent thrombolytic therapy shows partial filling defects in the pulmonary artery. (**F**): After 4 months’ treatment, there are some band-like enhancements (arrow) in the thrombus, indicating thrombolysis. LV—left ventricle; RV—right ventricle.

Pulmonary thromboembolism (PTE), which arises from venous thrombi occluding the pulmonary arteries, is the most common disease blocking the pulmonary lumen [10]. Acute PTE is fatal as it is associated with right ventricular dysfunction such as arrhythmia, hemodynamic collapse, and shock [11]. Computed tomography pulmonary angiography (CTPA) is the current reference method for its diagnosis. CPTA shows complete or partial filling defects in pulmonary arteries (Figure 4). Complete filling defects show no enhancement of the entire lumen without distention, unlike tumor embolism and pulmonary artery sarcoma. Partial filling defects can be centrally and peripherally located in the vessel and surrounded by contrast, showing a “railway track” sign on longitudinal images or forming acute angles with the artery wall [12]. Peripheral pulmonary infarction, producing peripheral wedge-shaped non-enhanced density in the lung parenchyma, is the most significant indirect sign (Figure 4).

**Figure 5:**
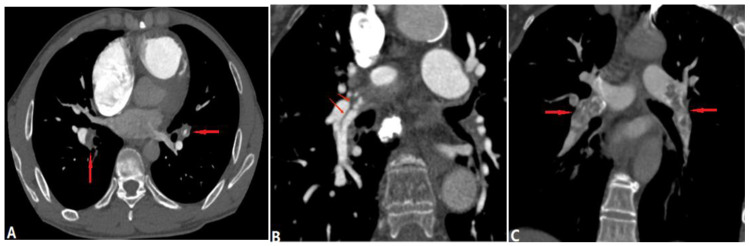
Chronic pulmonary embolism. (**A**): Axial CT image shows bilateral eccentric laminated filling defects and pulmonary artery stenosis (arrows) in a patient with chronic pulmonary embolism. (**B**): Coronal reconstruction CTA image in another patient with dyspnea shows a web (arrows) or band within a lower-right lobar pulmonary artery, consistent with chronic PE. (**C**): Coronal reconstruction CTA image in a patient with a history of acute PE shows calcified obstructive thrombus (arrows) within bilateral pulmonary arteries. CTA—computed tomography angiography; PE—pulmonary embolism.

Chronic PTE is characterized by chronic thrombotic remains in the pulmonary arteries after acute PTE, which can lead to chronic thromboembolic pulmonary hypertension (CTEPH)—a potentially life-threatening disease [13]. The remaining embolic material is incorporated into the vessel wall and covered by a thin layer of endothelial cells. The filling defects of chronic PTE on CTPA include the following: (a) retracted thrombi that present complete filling defects in the stenosed PA with an abrupt cutoff or narrowing of the vessel; (b) organized thrombi that show peripheral-lined filling defects adherent to the vessel wall, forming obtuse angles and occasionally containing calcifications; (c) recanalized thrombi that present contrast material traversing the arteries, with thickened walls forming intraluminal webs or bands (Figure 5). As the affected arteries are stenotic or occlusive, the vessels distal to the embolus have a smaller caliber, distinguishing them from acute PTE with normal or expanded vessels [14], but the MPA is widened. The secondary signs of chronic PTE include mosaic attenuation in the lungs and abnormal enlargement of the bronchial and systemic collateral vessels [15].

In CT(A), an enlarged PA diameter (PA diameter ≥ 30 mm, or a PA-to-aorta ratio > 0.9) and enlarged right heart chamber (RV:LV ratio ≥ 1) or septal deviation ≥ 140° suggest the presence of PH (Figure 4), which has a poorer prognosis in acute PE patients.

#### 4.1.2. In Situ Pulmonary Artery Thrombosis (PAT)

**Figure 6:**
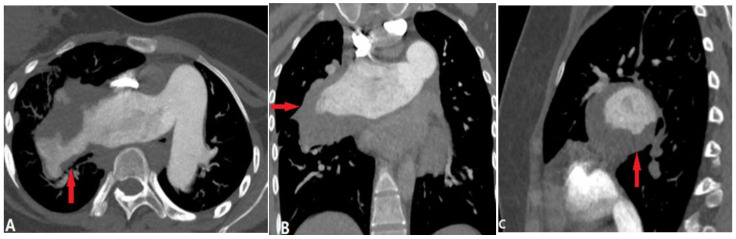
In situ thrombosis in pulmonary hypertension in a 16-year-old girl with primary pulmonary hypertension. (**A**,**B**): Axial and coronal CTA images show concentric, broad-based filling defects within the massively dilated right pulmonary artery, and the lumen (blood filling) is narrowed locally (arrow). The main and the left pulmonary arteries are also enlarged. (**C**): Cross-section of left pulmonary artery in MPR imaging shows interface irregularities (arrow) of the thrombosis within the symmetric widening and smooth outline of the vessel, which rule out compression caused by mass effect. CTA—computed tomography angiography; MPR—multiplanar reformation.

In situ pulmonary artery thrombosis (PAT) refers to de novo thrombosis of the pulmonary arterial system, associated with pulmonary infections, hypoxia, trauma, radiation therapy, pulmonary artery catheter, aberrant pulmonary structures (e.g., the pulmonary artery stump following pneumonectomy), severe pulmonary arterial hypertension, genetic mutations, and immunological and hematological systemic diseases [16]. These factors lead to pulmonary vascular endothelial cell injury, which triggers the coagulation cascade and induces thrombosis. These can also result in intimal hyperplasia, vascular remodeling, and even stenosis-flowing hemodynamic factors. Although they may be less important for the initiation of PA thrombosis, they affect vascular wall permeability and lead to thrombosis progression [17]. On CPTA, a thrombus in proximal PA is universally non-occlusive and eccentric, has obtuse angles to the vessel wall, and demonstrates a lack of vascular distention [18] (Figure 6). However, de novo thrombosis in sub-segments down to small PAs of 2–5 mm in diameter is difficult to detect on CTPA.

#### 4.1.3. Pulmonary Tumor Embolism and Pulmonary Tumor Thrombotic Microangiography (PTTM)

**Figure 7:**
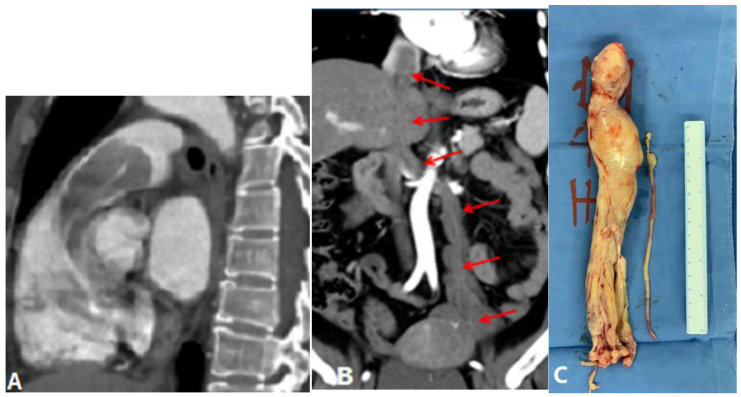
Tumor embolism (benign). (**A**): Sagittal MPR image in a patient with intravenous leiomyomatosis shows tumor embolism in the main pulmonary artery. (**B**): Coronal MPR image shows that the tumor originates from the uterus, along the left ovarian vein–left renal vein–inferior vena cava–right atrium–right ventricle, extending into the pulmonary artery (arrows). (**C**): Photograph of the resected tumor. MPR—multiplanar reformation.

**Figure 8:**
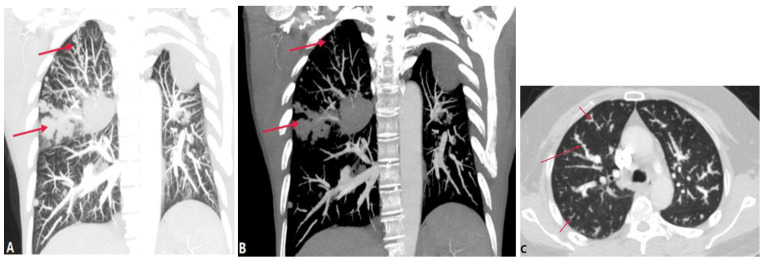
Tumor embolism and PTTM. (**A**): Coronal CT MPR image of a patient with right-lung cancer shows tumor emboli in the sub-segmental pulmonary arterial branches (arrows) and lumen occlusion. (**B**): Coronal CT scan image at the same level with soft tissue windowing shows the vascular dilatation and beading of sub-segmental arteries of the right pulmonary artery (arrows). (**C**): CT in another patient with a right atrium tumor shows tumor emboli with a tree-in-bud appearance within the secondary lobule arteries (short arrow) and beading of sub-segmental arteries (long arrow), which indicates PTTM.

Tumor embolism in the pulmonary artery is a rare clinicopathological entity characterized by the occlusion of pulmonary vessels by tumor clots. This can occur via three pathways: (a) direct extension of the thrombus from the systemic to the pulmonary vasculature; (b) tumor in the proximal arteries (macroembolism); (c) tumor in the small vessels (microembolism) [19]. The direct extension of tumor thrombus presents enhanced filling defects in PAs with or without lumen dilation on CTPA, distinguishing it from acute PE (Figure 7). PTTM is an advanced form of PTE, where tumor cells nest in the small vessels and lymphatic channels, triggering vessel endothelium proliferation and the coagulation cascade [20]. Owing to pulmonary microvascular disease and lymphangitic carcinomatosis, centrilobular nodules, tree-in-bud signs, peribronchovascular ground-glass opacities, and interlobular septal thickening can be seen on HRCT and CTPA [21] (Figure 8). For suspicious cases, maximum-intensity projection (MIP) is recommended to show the enlargement of pulmonary vasculature and small tumor embolus (Figure 8). With the occlusion of pulmonary vessels, PTTM can rapidly progress to PH and hypoxemia, or even death.

#### 4.1.4. Septic Pulmonary Embolism (SPE)

**Figure 9:**
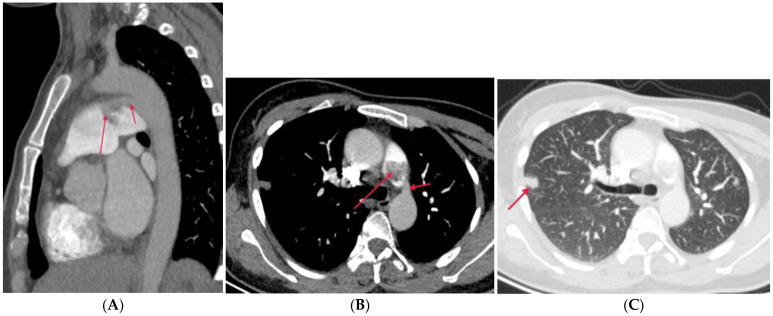
Septic pulmonary embolism in a 29-year-old woman with PDA and infective endocarditis. (**A**,**B**): Sagittal and axial CTPA images demonstrate a filling defect within the main pulmonary artery (long arrow) and at the end of the PDA (short arrow). (**C**): Axial CTPA on lung windowing shows sub-pleural opacity, consistent with peripheral infarction (arrow), which may be caused by the septic emboli. CTPA—computed tomography pulmonary angiography; PDA—patent ductus arteriosus.

Septic pulmonary embolism is an uncommon and challenging disorder that can cause the cross-sectional area of the pulmonary arteries to decrease. SPE thromboses containing micro-organisms are implanted into the PAs, not only obstructing the vessels but also leading to lung infarction and focal abscesses. It is associated with tricuspid valve endocarditis, septic thrombophlebitis, infected central venous catheters, heart pacemakers, and immunocompromised patients. The main pathogens include Gram-negative bacteria, Gram-positive bacteria, and fungi [22]. Typical characteristics of SPE are filling defects inside pulmonary arteries and thromboses often situated at turbulent-flow or slow-flow sites, which can be found in PDA (Figure 9). Associated lung abnormalities include subpleural and wedge-shaped infiltrates with peripheral enhancement and occasional cavitation, sometimes with a visible feeding vessel [23,24] (Figure 9). SPE symptoms are not specific, mostly present as bacteremia, dyspnea, chest pain, coughing, and other respiratory symptoms [25].

#### 4.1.5. Pulmonary Embolism Caused by Foreign Bodies

**Figure 10:**
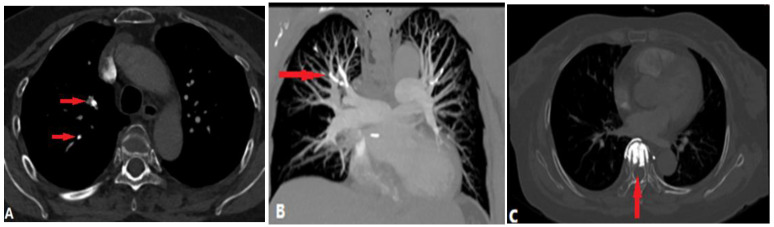
Pulmonary cement embolism. (**A**,**B**): Axial CTPA and coronal MIP show the cement embolus (high density-arrows) in the pulmonary arteries of the upper-right and -left lobes. (**C**): Cement (arrow) in the vertebral body and the external vertebral venous plexuses. CTPA—computed tomography pulmonary angiography; MIP—maximum-intensity projection.

**Figure 11:**
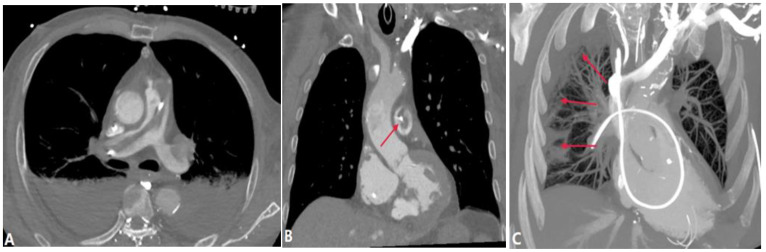
Catheter-related thrombosis in a patient post-CABG. (**A**). Axial CTA image shows filling defects extending from the main pulmonary to the right pulmonary artery along with the catheter. (**B**). Coronal CTA image shows the thrombus wrapped around the catheter (arrow). (**C**). Maximum-intensity projection image shows the path of the catheter and the peripheral consolidation, which is consistent with pulmonary infarction (arrows). CABG—coronary artery bypass grafting; CTA—computed tomography angiography.

Foreign body-induced pulmonary embolism is a stenosis or occlusion of the pulmonary vasculature by various organic and inorganic material blockages, such as air, catheter, cement (polymethylmethacrylate), metallic mercury, talc, fat, silicone, etc. [26]. In contrast to the clinical symptoms of PTE, the pathophysiologic effects are not only mechanical but also a consequence of the offending material’s nature, which may trigger an inflammatory cascade that causes deteriorating vascular, pulmonary, and cardiac function [27]. Cement injection within a vertebral body is widely used in patients with vertebral fracture and kyphosis, which may cause PE via the external vertebral venous plexuses (Figure 10). CT may show tubular areas of branching linear opacities filling the distal pulmonary artery branches. Catheter embolism is one of the most common iatrogenic causes of embolism. Fragments of central venous catheters, venous port catheters, guidewires, and vascular sheaths may reach and occlude the pulmonary arteries due to catheter tear, most often when catheters are removed [28]. CTPA often shows the catheter in the pulmonary artery (Figure 11). Foreign body-induced pulmonary embolism usually has a contact history, which can assist in the diagnosis.

#### 4.1.6. Pulmonary Artery Sarcoma

**Figure 12:**
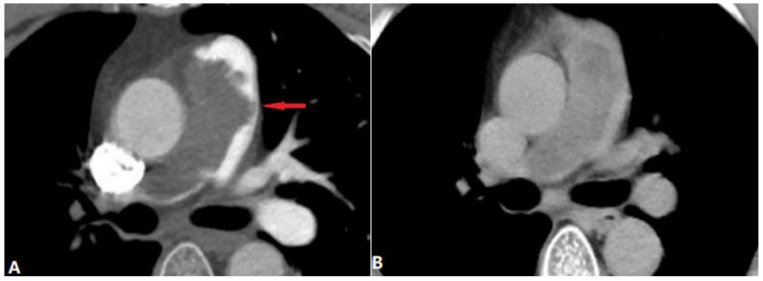
Pulmonary artery sarcoma with extensive involvement of the main pulmonary artery and the right trunk. (**A**): Contrast-enhanced CT showing a soft tissue mass filling the main and left pulmonary arteries (arrow). (**B**): The filling defects show delayed enhancement.

Pulmonary artery sarcoma (PAS) is a rare and lethal malignant tumor that arises from the intimal layer of the pulmonary artery, which is frequently misdiagnosed as pulmonary thromboembolism (PTE) with a poor prognosis. Early and correct diagnosis offers the only chance for survival. However, the clinical symptoms of PAS are always insidious—patients present with general cardiovascular or pulmonary symptoms, such as fever, anemia, weight loss, chest pain, cough, dyspnea, and syncope [29]. The distinguishing clinical features are unexplained pulmonary hypertension symptoms, a lack of risk factors for PTE, or poor response to anticoagulation [30]. Computed tomographic pulmonary angiography (CTPA) is useful in clinically suspicious cases of PAS. The typical feature of PAS on CTPA imaging is that the lesions always involve the main PA, which can extend into the main pulmonary trunk and right ventricular outflow tract, pulmonary artery trunk, or segmental PAs. It tends to occupy the entire lumen with local aneurysmal dilatation, which means that PAS grows outside the artery. Other features include delay enhancement, wall eclipsing sign, and proximal lobulated and bulging margins [31] (Figure 12). The “wall eclipsing sign” and enhancement can be helpful in distinguishing PAS from thromboembolic disease [32].

### 4.2. Vessel Wall Lesions

Vasculitis 

Vasculitis is a diverse group of diseases characterized by inflammation within and around blood vessel walls, affecting large, medium, and small vessels.

#### 4.2.1. Takayasu Arteritis (TA)

**Figure 13:**
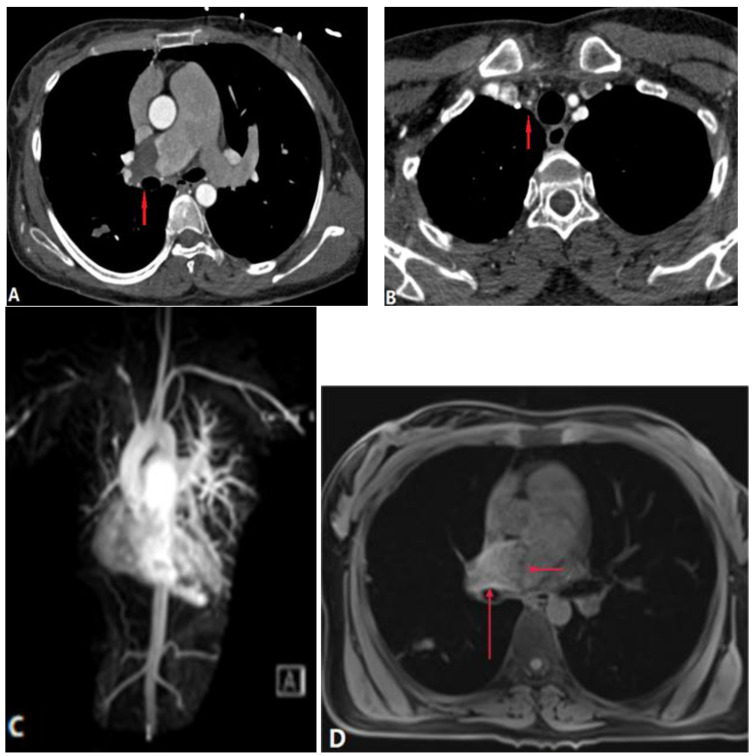
Vasculitis-related pulmonary artery thrombosis. Takayasu arteritis in a 33-year-old woman with dizziness and chest compress. (**A**): Contrast-enhanced axial CT demonstrates a filling defect and occlusion of the right pulmonary artery wall (arrow), and the main pulmonary is dilated. (**B**): Axial CT image shows the thickening walls of the right subclavian artery and right common carotid artery, and the lumen is narrow (arrow). (**C**): Coronal MIP contrast-enhanced MRA image shows that the right brachiocephalic artery, right subclavian artery, and right common carotid artery are stenosed or occluded; the right pulmonary artery and distal segment are stenosed. (**D**): Axial MR T1 image shows mild thickening of the right pulmonary artery (long arrow) and iso-intensity thrombosis (short arrow). MIP—maximum-intensity projection; MRA—magnetic resonance angiography.

Takayasu arteritis (TA) is an autoimmune large-vessel vasculitis of unknown etiology that typically involves the aorta and its main branches, and pulmonary artery lesions are not uncommon. The disease is most prevalent among Asian females under 40 years of age [33]. Pulmonary artery involvement usually occurs in late-stage TA, and the main clinical presentations are dyspnea, chest pain, hemoptysis, and coughing [34]. The pathology is characterized by the involvement of all arterial layers with variable inflammatory infiltration [35]. The most common imaging manifestations of pulmonary artery involvement are stenosis, followed by occlusion and arterial wall thickening [34] (Figure 13). CTPA displays thickened and enhanced vascular walls during the inflammatory phase, and symmetric smoothly tapered areas of circumferential narrowing near the origin of the primary branches of the aorta, often with copious collateral vessels in the occlusive phase [33]. Other than PA stenosis, pulmonary infarction is also occasionally shown on HRCT. The clinical presentation of TA with pulmonary artery involvement is different from classical TA, which may lead to misdiagnosis and inappropriate treatment, even PH. It is crucial for radiologists to recognize these findings [35].

#### 4.2.2. Behçet Disease (BD)

**Figure 14:**
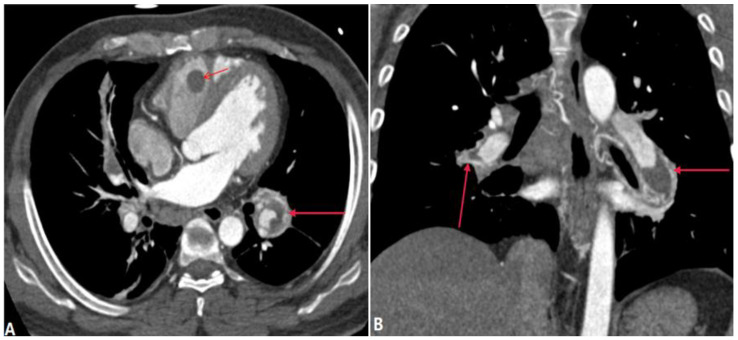
Vasculitis-related pulmonary artery thrombosis. Behcet syndrome (10 years) in a 40-year-old man with hemoptysis. (**A**,**B**): Axial and coronal CT MPR images show aneurysmal dilatation of the bilateral lower lobe pulmonary artery with wall thickening and enhancement (long arrows), in situ thrombosis, and stenosis or occlusion. In situ thrombosis can also be found in the right ventricle (short arrow in (**A**)). MPR—multiplanar reformation.

Behçet disease (BD) is a rare multisystemic and chronic inflammatory disorder with an unknown cause. The clinical features of the disease are recurrent oral and genital ulcers, and uveitis. The main pathologic process of BD is vasculitis and perivascular infiltration affecting vessels of various sizes, thus, the characteristic manifestation of pulmonary artery involvement is pulmonary arterial aneurysm. Due to the vascular inflammation, in situ thrombus is commonly detected, which causes stenosis of the distal vessels (Figure 14). CTPA is an important diagnostic imaging technique for patients with BD. The CTPA imaging features of BD are the dilatation of the pulmonary artery lumen with thrombosis, stenosis of the distal vessels, enhancement of thickened arterial walls, and formation of collateral circulation [36]. Although TA and BD are both vasculitis, pulmonary artery stenosis is relatively common in TA compared to BD. According to age, gender, and clinical features, it is not difficult to distinguish these conditions.

#### 4.2.3. Swyer–James–Macleod Syndrome (SJMS)

**Figure 15:**
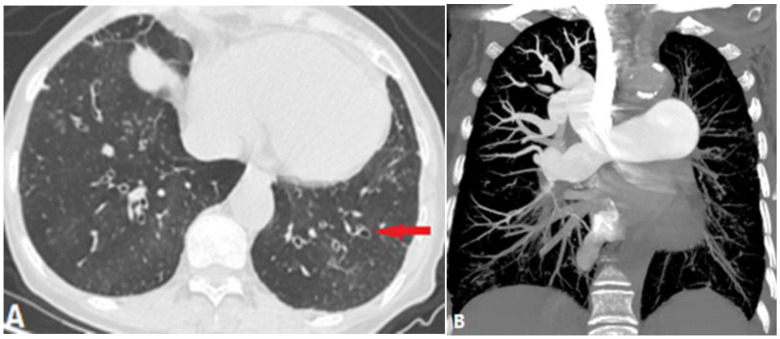
SMJS. A 71-year-old woman with Swyer–James–Macleod syndrome. (**A**): Lung window axial CT shows bronchiectasis and infections in the lower-right and -left lobes (arrow), and the hyperlucent right lung. (**B**): Coronal MIP of CTPA shows hypoplasia of the pulmonary vasculature of the left lung. CTPA—computed tomography pulmonary angiography; MIP—maximum-intensity projection.

Swyer–James–MacLeod syndrome (SMJS) is a rare disease characterized by pulmonary artery hypoplasia, unilateral hyperlucency, and bronchiectasis, which is believed to be the result of childhood bronchiolitis obliterans. SJMS is often diagnosed in childhood with chronic pulmonary symptoms, although some patients are diagnosed only in adulthood [37]. The most common clinical symptoms of SJMS are coughing, recurrent pulmonary infections, decreased exercise capacity, and hemoptysis. Unilateral hyperlucency can be found on radiographs. Chest CT may demonstrate emphysema, bullae, bronchiectasis, atelectasis, and scarring. CTPA can demonstrate hypoplasia of the pulmonary vasculature in the affected lung or segment (Figure 15). SJMS diagnosis is based on clinical symptoms and imaging findings. Histopathology is not essential but further supports the diagnosis, presenting emphysema and cystic cavities in pathological sections [37].

#### 4.2.4. Arterial Dissection (AD)

**Figure 16:**
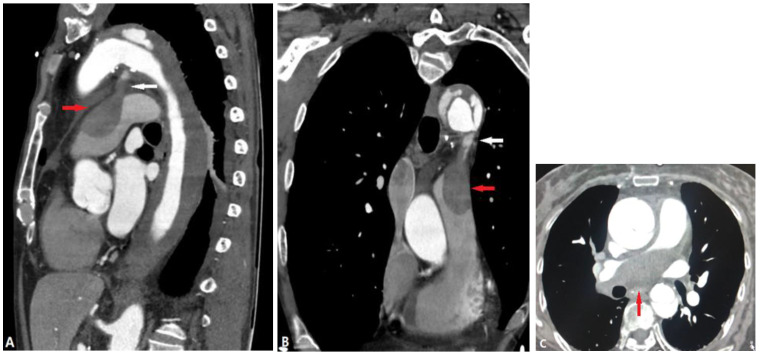
Aortic dissection. (**A**,**B**): Sagittal and coronal CTA images show the hematoma of an aortic dissection extending to the pulmonary artery via the PDA (white arrow), which causes PA stenosis (red arrow). (**C**): Another aortic dissection patient presents with right pulmonary artery stenosis (red arrow) because of the discontinuity of the media. CTA—computed tomography angiography; PDA—patent ductus arteriosus.

Arterial dissection (AD) is a lethal condition caused by elevated blood pressure leading to the separation of the media layers [38], followed by the formation of a false lumen within the arterial wall. Several primary conditions are known to be linked to elevated pulmonary arterial wall stress, such as primary hypertension, patent ductus arteriosus (PDA), etc., which lead to pulmonary AD and stenosis [39] (Figure 16). There are also some secondary conditions. For instance, during dissection, discontinuity of the media may involve the pulmonary artery due to the continuous adventitia of the aorta and pulmonary artery, which can mimic pulmonary embolism and can be lethal. Without proper treatment, rupture of the aortic dissection can develop into mediastinal hematoma, which presses the pulmonary arteries. In other cases, pulmonary AD may occur through the PDA, which causes adventitia–media separation followed by an intramural hematoma (IMH) [39].

### 4.3. Extra-Luminal Abnormalities

#### 4.3.1. Fibrosis Mediastinitis (FM)

**Figure 17:**
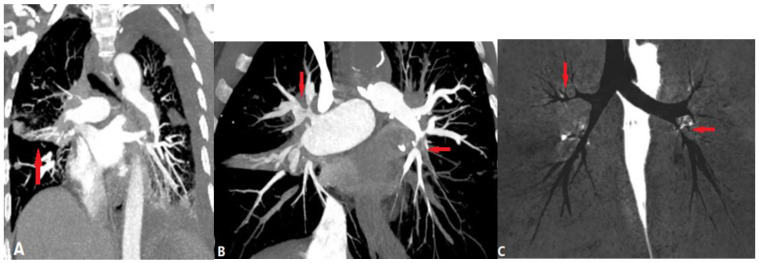
TB-FM. (**A**): Coronal MIP shows the dense and calcified tissues wrapping the pulmonary vasculatures, causing stenosis and atelectasis of the right middle lobe (arrow). (**B**,**C**): The coronal MIP and MinIP show the same segment of the bronchus and pulmonary vessels (arrows) affected by the tissues. MIP—maximum-intensity projection; MinIP—minimum-intensity projection.

**Figure 18:**
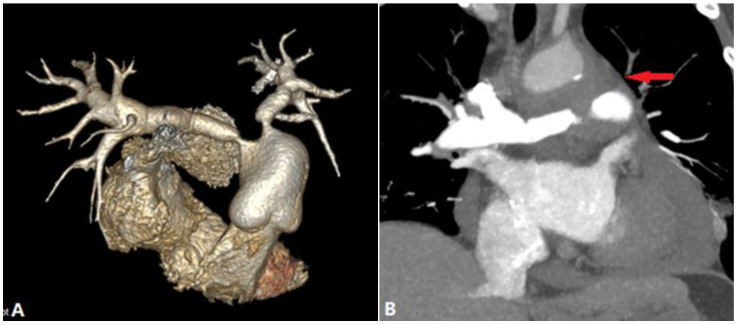
IgG4-related FM. Three-dimensional volume rendering image (**A**) of pulmonary arteries. (**B**) MIP shows the stenosed proximal segment of the right and left pulmonary arteries caused by the infiltrative soft tissue, without calcification (arrow). MIP—maximum-intensity projection.

Fibrosis mediastinitis (FM) is a benign but lethal condition caused by fibrous tissue proliferation in the mediastinum with encasement of the mediastinal viscera and compression of mediastinal bronchovascular structures, therefore, pulmonary hypertension is frequently observed. FM is divided into two sub-types: the granulomatous sub-type and the non-granulomatous sub-type. The former is usually associated with some infectious or inflammatory conditions, such as tuberculosis (the most common cause in China) or *Histoplasma capsulatum* (the most common cause in the United States). The latter type is less common, diffuse-located, and more related to autoimmune diseases [39]. The most common clinical symptoms include cough, dyspnea, hemoptysis, and pleuritic chest pain. The FM triad of the granulomatous sub-type includes atelectasis, pleural effusion, and prominent right-heart border, which is the most typical feature on chest X-ray and CT [40]. Contrast-enhanced CT is the modality of choice for evaluating patients with suspected FM. The major body of the fibrous tissues (often with calcification) locally wrapping the same segment of the bronchus and pulmonary vessels is mildly enhanced on CTPA and causes the stenosis of affected pulmonary vessels (Figure 17), which is the critical point to distinguish from other diseases (tumor or vasculitis). In contrast, the non-granulomatous sub-type often shows diffused, non-calcified soft tissues around the stenosed pulmonary arteries (Figure 18).

#### 4.3.2. Tumors Causing Pulmonary Artery Stenosis

**Figure 19:**
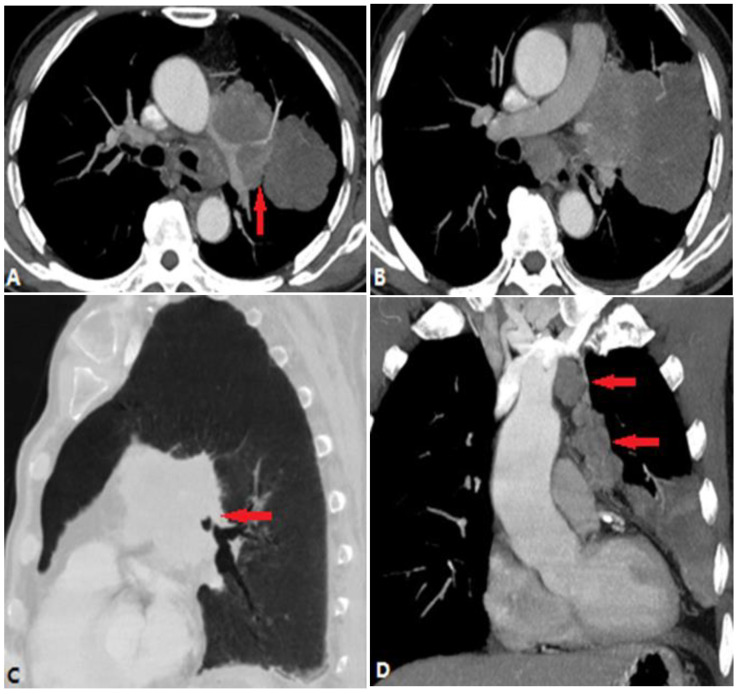
Tumor in a 50-year-old male with lung cancer. (**A**,**B**): Axial MIP shows that the tumor infiltrates and compresses the pulmonary arteries of the left lung, following the lumen narrowing (arrow). (**C**): Sagittal view shows the occluded bronchus (arrow). (**D**): Enlarged lymph nodes (arrows) on coronal CT due to metastasis. MIP—maximum-intensity projection.

Lung metastatic tumors, primary bronchogenic neoplasia, and other tumors may locally invade and obliterate pulmonary arteries. Tumors can invade and grow along pulmonary vessels farther than their lung interface, which can completely obliterate the affected vessel. Tumors can also compress the pulmonary vessels because of the mass effect. The pulmonary artery is one of the most invaded thoracic vessels in patients with advanced lung cancer, which can lead to life-threatening massive bleeding [41]. CTPA is useful in finding tumors to suggest a diagnosis. In CPTA, primary lung cancer often demonstrates compression and narrowing of the pulmonary artery (Figure 19). CT features include wall thickening and lumen narrowing without occlusion, which are valuable for diagnosis and making surgical plans.

#### 4.3.3. Aortic Aneurysm

**Figure 20:**
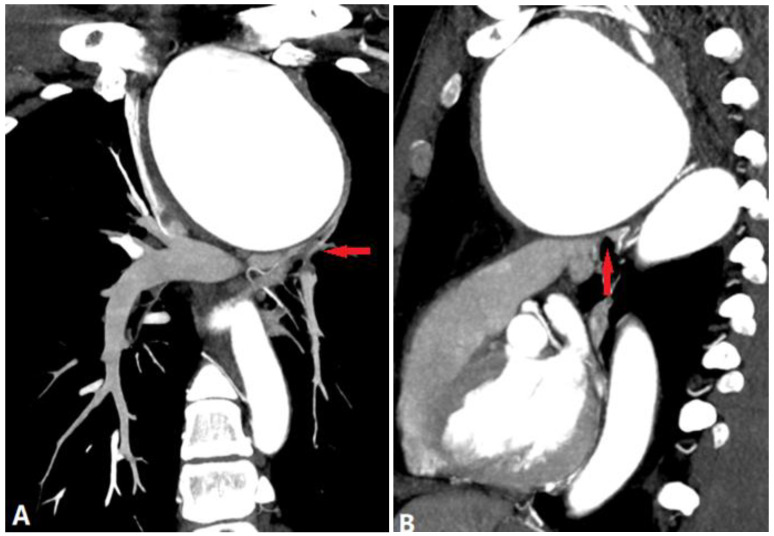
Aortic aneurysm. (**A**,**B**): Coronal and sagittal MIP images show the direct compression of the left pulmonary arteries by the giant aortic aneurysm (arrow). MIP—maximum-intensity projection.

Direct compression of the pulmonary artery is a rare complication of a giant aortic aneurysm via a mass effect, which can cause pulmonary artery stenosis and even right-heart failure [42]. A large aortic aneurysm compressing the adjacent pulmonary artery can be seen on CTA (Figure 20). With the elevated volume and pressure, RV enlargement is also shown. Aortic surgery is needed, and CTA makes it easier to understand the anatomy and make a proper surgical plan [43].

## 5. Complications of Radiofrequency Ablation of Atrial Fibrillation

**Figure 21:**
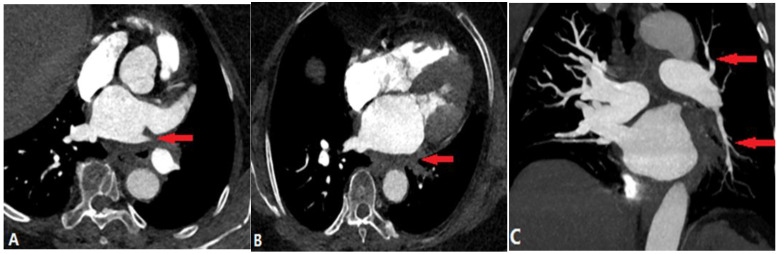
Radiofrequency ablation of atrial fibrillation. (**A**): Axial CTA shows beak-like narrowing of the left superior pulmonary vein (arrow). (**B**): Axial CTA shows the occluded inferior pulmonary vein (arrow). (**C**): Coronal MIP shows the narrowing of left pulmonary arteries due to decreased blood flow (arrows). MIP—maximum-intensity projection.

The complications of radiofrequency ablation of atrial fibrillation are pulmonary vein stenosis, pulmonary vein occlusion, and lung infarction [44]. Furthermore, it causes the pulmonary artery on the affected side to narrow. The mechanism may be the ectopic foci of abnormal electrical activity in the ostia or muscular sleeves of the pulmonary veins, which are the target of ablation [45]. The clinical symptoms are similar to some respiratory diseases, such as dyspnea on exertion of escalating intensity, pleuritic chest pain, hemoptysis, persistent cough, and fever. The gold diagnostic standard is cardiac catheterization showing venous narrowing or occlusion [46]. As a non-invasive and efficient tool, CTPA shows direct or indirect evidence of occlusion (Figure 21). These findings include septal thickening in the upper-left lobe, increased attenuation in the mediastinal fat adjacent to the left superior pulmonary vein, and localized lymphadenopathy [44].

## 6. Conclusions

Pulmonary artery stenosis or occlusion can be caused by a variety of conditions, with high mortality rates in some cases. However, its clinical manifestation is often non-specific and insidious. CTPA can make a definitive diagnosis in the majority of the causative diseases and detect other associated conditions. It is important to recognize and classify these pulmonary vascular diseases by analyzing appropriate imaging features, as this is helpful for early diagnosis and interventions. This is especially important in the emergency department, since an accurate clinical evaluation is crucial, together with diagnostic imaging, to reach a prompt diagnosis.

## Figures and Tables

**Table 1:** Causes of pulmonary artery stenosis.

Congenital Disease	Manuscript Location	
	Section 3.1	Pulmonary valvular stenosis
	Section 3.2	Pulmonary artery atresia
	Section 3.3	Pulmonary vein atresia
Acquired disease		
Intraluminal anomalies		
	Section 4.1.1	Pulmonary thromboembolism
	Section 4.1.2	In situ pulmonary artery thrombosis
	Section 4.1.3	Pulmonary tumor embolism
	Section 4.1.3	Pulmonary tumor thrombotic microangiography
	Section 4.1.4	Septic pulmonary embolism
	Section 4.1.5	Foreign bodies pulmonary embolism
	Section 4.1.6	Pulmonary artery sarcoma
Vessel wall lesions		
	Section 4.2.1	Takayasu arteritis
	Section 4.2.2	Behçet disease
	Section 4.2.3	Swyer–James–Macleod Syndrome
	Section 4.2.4	Arterial dissection
Extraluminal anomalies		
	Section 4.3.1	Fibrosis mediastinitis
	Section 4.3.2	Tumor
	Section 4.3.3	Aortic aneurysm
	Section 5	Complications of radiofrequency ablation of atrial fibrillation

## Data Availability

Data are not available.
